# Expression of XCR1 Characterizes the Batf3-Dependent Lineage of Dendritic Cells Capable of Antigen Cross-Presentation

**DOI:** 10.3389/fimmu.2012.00214

**Published:** 2012-07-18

**Authors:** Annabell Bachem, Evelyn Hartung, Steffen Güttler, Ahmed Mora, Xuefei Zhou, Anika Hegemann, Maud Plantinga, Elisa Mazzini, Patrizia Stoitzner, Stephanie Gurka, Volker Henn, Hans W. Mages, Richard A. Kroczek

**Affiliations:** ^1^Molecular Immunology, Robert Koch-InstituteBerlin, Germany; ^2^Laboratory of Immunoregulation and Mucosal Immunology, Department of Respiratory Diseases, University Hospital of GhentGhent, Belgium; ^3^Department of Experimental Oncology, European Institute of OncologyMilan, Italy; ^4^Department of Dermatology and Venereology, University of InnsbruckInnsbruck, Austria

**Keywords:** dendritic cells, XCR1, Batf3, cross-presentation, lineage marker

## Abstract

Cross-presentation of antigen by dendritic cells (DCs) to CD8^+^ T cells is a fundamentally important mechanism in the defense against pathogens and tumors. Due to the lack of an appropriate lineage marker, cross-presenting DCs in the mouse are provisionally classified as “Batf3-IRF-8-Id2-dependent DCs” or as “CD8^+^ DCs” in the spleen, and as “CD103^+^CD11b^−^ DCs” in the periphery. We have now generated a mAb to XCR1, a chemokine receptor which is specifically expressed on CD8^+^ DCs and a subpopulation of double negative DCs in the spleen. Using this antibody, we have determined that only XCR1^+^CD8^+^ (around 80% of CD8^+^ DCs) and their probable precursors, XCR1^+^CD8^−^ DCs, efficiently take up cellular material and excel in antigen cross-presentation. In lymph nodes (LNs) and peripheral tissues, XCR1^+^ DCs largely, but not fully, correspond to CD103^+^CD11b^−^ DCs. Most importantly, we demonstrate that XCR1^+^ DCs in the spleen, LNs, and peripheral tissues are dependent on the growth factor Flt3 ligand and are selectively absent in *Batf3*-deficient animals. These results provide evidence that expression of XCR1 throughout the body defines the Batf3-dependent lineage of DCs with a special capacity to cross-present antigen. XCR1 thus emerges as the first surface marker characterizing a DC lineage in the mouse and potentially also in the human.

## Introduction

In the immune system, dendritic cells (DCs) are specialized on the uptake and presentation of antigens to T cells. In the absence of DC-specific markers, mouse “conventional DCs” (cDCs) are being currently defined as lineage marker-negative hematopoietic cells expressing high levels of MHC class II and the integrin CD11c. By a number of criteria cDCs differ from “plasmacytoid DCs” (pDCs), which only have a limited potential to take up and present antigen (Villadangos and Young, 2008). In some antigen targeting studies, splenic cDCs are subdivided into mutually exclusive CD205^high^ and DCIR2^+^ populations, the latter recognized by mAb 33D1 (Dudziak et al., 2007). More commonly, cDCs are classified into CD4^+^ DCs (around 60% of DCs), CD8^+^ DCs (20%, encompassing almost all CD205^high^ DCs), and CD4^−^CD8^−^ DCs (DN DCs, 20%; Vremec et al., 2000). These subdivisions allowed in the past to recognize major differences between DC subsets, both in their ontogeny and function. It was demonstrated that splenic CD8^+^ DCs critically depend in their development on the growth factor Flt3 ligand (Maraskovsky et al., 1996; McKenna et al., 2000) and the transcription factor (TF) Batf3 (Hildner et al., 2008). Functionally, CD8^+^ DCs were demonstrated to be particularly efficient in the uptake of cells stressed by (intracellular) infection (Iyoda et al., 2002; Schulz and Reis e Sousa, 2002; Schnorrer et al., 2006). Moreover, CD8^+^ DCs have consistently been shown to excel in antigen “cross-presentation,” in which antigen is not presented in the context of MHC class II to CD4^+^ T cells, but instead shunted to the MHC class I pathway and presented to CD8^+^ T cells (den Haan et al., 2000; Pooley et al., 2001). Thus, splenic CD8^+^ DCs were until recently regarded as a coherent population of DCs closely cooperating with CD8^+^ T cells in the surveillance of stressed/transformed cells for “danger” (Shortman and Heath, 2010).

The equivalents of “classical CD8^+^ DCs” in the periphery of the mouse immune system were not easily identifiable since CD8 is only expressed to a low degree on some DC subpopulations in peripheral organs. Several lines of evidence indicated that in the periphery CD103^+^CD11b^−^ DCs largely correspond to the “classical CD8^+^ DCs,” but the use of CD103 and CD11b, even in combination with other markers, did not allow a consistent delineation of these DCs (Shortman and Heath, 2010; Hashimoto et al., 2011). Recently, it became apparent that both the “classical splenic CD8^+^ DCs” and also their counterparts in the periphery are critically dependent on the TFs Batf3, IRF-8 (also designated ICSBP), and Id2 (Schiavoni et al., 2002; Aliberti et al., 2003; Hacker et al., 2003; Hildner et al., 2008; Ginhoux et al., 2009; Edelson et al., 2010). Since this TF-dependence appeared to be a consistent feature for the cross-presenting DCs in the entire body, they were also termed “Batf3-IRF-8-Id2-dependent DCs” (Hashimoto et al., 2011).

Using a reporter system, we have recently recognized that the chemokine receptor XCR1 is exclusively expressed in splenic CD8^+^ DCs, in a subset of DN DCs, and in corresponding DCs in peripheral lymphoid tissues, but not in other cell types in the mouse (Dorner et al., 2009). Similar results were independently obtained by the group of Dalod (Crozat et al., 2010, 2011). Since antibodies detecting the XCR1 protein were not available until now, all observations on the specific expression of XCR1 in CD8^+^ DCs were based on XCR1-reporter mice and extensive qPCR analyses of purified cell populations.

We now have generated a mAb which specifically detects the murine XCR1 receptor. This allowed us to follow up our earlier observations that only around 80% of splenic CD8^+^ DCs express XCR1 at mRNA level (Dorner et al., 2009). In our present report we show that regarding XCR1 cell surface expression, splenic CD8^+^ DCs are a heterogeneous population, with only XCR1^+^CD8^+^ DCs (but not XCR1^−^CD8^+^ DCs) capable of antigen cross-presentation. We further demonstrate that XCR1^+^DN DCs resemble XCR1^+^CD8^+^ DCs in their phenotype and function, and are apparently precursors of XCR1^+^CD8^+^ DCs. Our results show that XCR1^+^ DCs and CD103^+^CD11b^−^ DCs are largely congruent populations in lymph nodes (LNs) and peripheral organs, but additional XCR1^+^ DCs can be found, which do not express CD103. Most importantly, we provide evidence that the DC populations expressing XCR1 in the spleen, LNs, and peripheral tissues are selectively absent in *Batf3*-deficient animals. Thus, expression of XCR1 uniformly characterizes a DC lineage known to excel in antigen cross-presentation, recognized until now as “classical CD8^+^ DCs,” “CD103^+^CD11b^−^ DCs,” or “Batf3-IRF-8-Id2-dependent DCs.” The use of XCR1 as a “functional” marker will facilitate the analysis of DC biology *in vivo*, also in the human, where XCR1 is expressed on a CD141^+^ DC population homologous to “classical CD8^+^ DCs” (Bachem et al., 2010; Crozat et al., 2010).

## Materials and Methods

### Mice and Flt3 ligand treatment

Unless indicated otherwise, 8- to 10-week-old C57BL/6 female mice were used for cell isolation and immunohistological analyses. CX3CR1^GFP^ (Jung et al., 2000), Lang-EGFP mice (Kissenpfennig et al., 2005), B6.XCR1-LacZ (The Jackson Laboratories), and *Xcl1*-deficient mice (Dorner et al., 2009) were on the C57BL/6 background. 129/Sv WT mice (129S2/SvPasCrl substrain) were purchased from Charles River. *Icsbp/Irf-8*-deficient (Holtschke et al., 1996) and *Batf3*-deficient mice (Hildner et al., 2008) were on the 129/Sv background. OT-I TCR-transgenic mice were crossed onto the B6.PL background to allow identification of CD8^+^ T cells using the CD90.1 marker. For Flt3 ligand treatment, C57BL/6 mice were injected with 1 × 10^6^ B16 cells secreting Flt3 ligand (Mach et al., 2000) in 100 μl PBS s.c. All mice were bred under specific pathogen-free conditions in the animal facility of the Federal Institute for Risk Assessment (Berlin, Germany). All animal experiments were performed according to state guidelines and approved by the local animal welfare committee.

### Antibodies

Hybridomas producing mAb recognizing CD4 (clone YTS 191.1), CD8 (53-6.72), CD11b (5C6), CD11c (N418), CD16/32 (2.4G2), CD19 (1D3), CD24 (M1/69.16.11.HL), CD45R/B220 (RA3-6B2), DCIR2 (33D1), Ly6G/C (RB6-8C5), MHC class II (M5/114.15.2), and NK1.1 (PK136) were obtained from ATCC, CD90.1 (OX-7) from ECACC. MAb to CD103 (M290), CD172a (P84) were from BD Biosciences, to CD69 (H1.2F3), CD317 (eBio927), and PD-1 (J43) from eBioscience. Anti-mClec9A/DNGR-1 antibodies 24/04-10B4 (Caminschi et al., 2008) and 1F6 (Sancho et al., 2008) were used. Anti-CD3 (KT3) was generously provided by H. Savelkoul, anti-CD25 (2E4) by E. Shevach, and anti-DEC205 (NLDC-145, CD205) by G. Kraal. Anti-ICOS (MIC-280) antibody was generated as described before (Löhning et al., 2003).

### Generation of the monoclonal anti-murine XCR1 antibody

Homozygous B6.XCR1-lacZ mice were five times immunized i.p. with 30 × 10^6^ CD11c-enriched cells from C57BL/6 WT mice in combination with heat-inactivated *B. pertussis* (Chiron Behring). One day after the last boost, splenocytes of the immunized mice were fused with the myeloma cell line P3 × 63Ag8.653 (ATCC) according to standard methods. The resulting hybridomas were screened for mAb against XCR1 by flow cytometry of DCs from C57BL/6 WT mice enriched by density gradient centrifugation, DCs from B6.XCR1-lacZ mice served as negative control. As secondary reagent, Cy5-AffiniPure Goat anti-Mouse IgG (Fcγ fragment specific; Jackson ImmunoResearch) was used. Screening of 1,500 hybridomas yielded one specific mAb against XCR1, which was designated MARX10 (IgG2b; determined by ELISA).

### Cell isolation

Splenocytes were obtained by mashing spleens through 70 μm cell sieves into PBS, followed by erythrocyte lysis with ACK Buffer (155 mM NH_4_Cl, 10 mM KHCO_3_, 0.1 mM EDTA). Where indicated, DCs were enriched by cutting spleens into small pieces followed by digestion with Collagenase D (500 μg/ml) and DNase I (20 μg/ml, both Roche) for 20 min at 37°C in RPMI 1640 containing 2% FCS (low endotoxin, Biochrom); EDTA (10 mM) was added for additional 5 min and cells were filtered through a 70-μm nylon sieve (BD Falcon). Low density cells were further enriched by centrifugation over a 1.073-g/ml density gradient (NycoPrep, Axis-Shield), followed by magnetic cell sorting with CD11c microbeads (Miltenyi Biotec). For isolation of gut DCs, the small intestine was freed from fat and Peyer’s patches, opened longitudinally, and stirred in PBS, 2% BSA, 1 mM EDTA, 1 mM DTT for 8 min at 37°C. After additional stirring under the same conditions without DTT, epithelial cells in solution were discarded, intestinal tissue was minced, and stirred in 500 μg/ml Collagenase VIII (Sigma) and 20 μg/ml DNAse I (Roche) for 45 min at 37°C. Low density cells were enriched by centrifugation over a 1.073-g/ml density gradient. Skin-draining LNs (pooled inguinal and axillar LNs) and mesenteric LNs were mashed through sieves and subjected to enzymatic digestion as described for splenic tissue. Lungs were perfused with 10 ml PBS through the right ventricle of the heart and separated from LNs. Lung tissue was cut into pieces, dissociated with the gentleMACS (Miltenyi Biotec), and digested for 30 min with 20 μg/ml Liberase TM and DNase I (20 μg/ml, both Roche) at 37°C in RPMI 1640 containing 2% FCS (low endotoxin, Biochrom); EDTA (10 mM) was added for additional 5 min to stop Liberase activity. After further dissociation with the gentleMACS, lung tissue was filtered through a 70-μm nylon sieve (BD Falcon) and erythrocytes were lysed with ACK Buffer.

### Flow cytometry and cell sorting

Antibodies were titrated for optimal signal-to-noise ratio. To block unspecific binding to Fc-receptors, cells were pre-incubated with 100 μg/ml 2.4G2 mAb for flow cytometry and in addition with 50 μg/ml purified rat Ig (Nordic) for flow sorting. Standard staining with mAb was in PBS, 0.25% BSA, 0.1% NaN_3_ for 20 min on ice, staining for Clec9A was in the same buffer for 20 min at 37°C. For exclusion of dead cells 4′,6-diamidino-2-phenylindole (DAPI) was added 5 min before measurement. Data were acquired on a LSR II flow cytometer (BD Biosciences), and analyzed using FlowJo (Tree Star, Inc.). Doublets and autofluorescent cells were excluded from the analysis. For lung stainings, CD45 was used to define lymphocytes. In all organs, DCs were defined as CD11c^+^MHC II^+^Lin^−^ cells. The lineage cocktail contained mAbs directed to CD3 and B220, a mAb to Ly6G/C was added for analyses of splenocytes. DCs isolated from LN were considered as migratory or resident based on their levels of MHC II expression. For flow sorting of splenic DCs, CD11c^+^MHC class II^+^Lin^−^ cells were stained with the respective antibodies and sorted based on their expression of CD8 and XCR1 on a FACSAriaII (BD Biosciences). For cell uptake experiments, 300-19-ΔOVA cells (Dorner et al., 2009) were labeled with CFSE (10 μM, 12 min, 37°C), washed, and injected i.v. (10 × 10^6^ cells in 200 μl PBS).

### Histology

For standard histological analysis, cryostat sections (12 μm) of spleens from C57BL/6 WT and B6.XCR1-lacZ (homozygous and heterozygous) mice were fixed in acetone for 10 min at RT. Endogenous peroxidase was inhibited with a blocking solution (PBS, 1 mM NaN_3_, 10 mM glucose, 1 U/mL glucose oxidase) for 1 h at 37°C. Unspecific binding sites were saturated with Casein Solution (Vector Laboratories) supplemented with mAb 2.4G2 (100 μg/ml) and rat IgG (50 μg/mL) for 1 h at RT. Sections were stained with DIG-coupled mAb MARX10 or FITC-conjugated mAb KT3 or RA3-6B2, washed, and incubated with anti-DIG Fab coupled to alkaline phosphatase or anti-fluorescein Fab coupled to horseradish peroxidase (both Roche). The stainings were developed using the Blue Alkaline Phosphatase Substrate Kit and the ImmPACT DAB Peroxidase Substrate (both Vector Laboratories) sequentially. For confocal microscopy, sections were stained with fluorophore-coupled antibodies and biotinylated mAb MARX10, the XCR1-staining was amplified with peroxidase-conjugated streptavidin (Jackson ImmunoResearch), followed by fluorescence-labeled tyramide (TSA-Kit, Molecular Probes). Nuclei were counterstained with DAPI (100 ng/ml) and the sections analyzed on a LSM 780 with ZEN2010 imaging software (Carl Zeiss).

### Cross-presentation assay

For *in vivo* loading with antigen, C57BL/6 mice were injected i.v. with either 2 mg OVA (Sigma-Aldrich) or 10 × 10^6^ 300-19-ΔOVA cells (Dorner et al., 2009) in 200 μl PBS. Before injection, LPS was removed from OVA using EndoTrap red (Hyglos) resulting in <0.5 EU of endotoxin per milligram of protein as determined by the LAL assay (Charles River). Twelve hours after injection with soluble or cell-associated antigen, DC subsets were sorted to high purity (>98.5%) as described in flow cytometry and cell sorting. OT-I CD8^+^ T cells were enriched by depleting OT-I splenocytes expressing CD4, CD11b, CD11c, B220, or NK1.1 using biotinylated mAb and anti-biotin magnetobeads (Miltenyi Biotec); after their resting state was confirmed using mAb directed to CD25, CD69, PD-1, and ICOS, they were labeled with CFSE (5 μM, 10 min, 37°C). For cross-presentation assays, 1 × 10^5^ CFSE-labeled OT-I CD8^+^ T cells were co-cultured with titrated numbers of DC subsets (1,000–30,000) in 200 μl RPMI medium containing 10% FCS, 50 μM 2-mercaptoethanol, and 100 μg/ml penicillin/streptomycin in 96-well round-bottomed plates (Nunc) for 2.5 days. Thereafter, proliferation of OT-I CD8^+^ T cells was determined in the CFSE dilution assay after gating on CD90.1 cells. As a positive control, sorted DC subsets from untreated mice were loaded with 1 μM of the OVA peptide SIINFEKL in medium for 1 h at 37°C, washed, and co-cultured with CFSE-labeled OT-I T cells (1 × 10^4^ DCs, 1 × 10^5^ T cells) for 2.5 days.

### Statistical analysis

All statistical analyses were performed with Prism software (GraphPad Software, Inc.). Differences between DC subsets were analyzed by paired Student’s *t*-test, a *p*-value of <0.05 was considered significant (*p < 0.05; **p < 0.01; ***p < 0.001).

## Results

### Generation of a monoclonal antibody directed to XCR1

**Figure 1:**
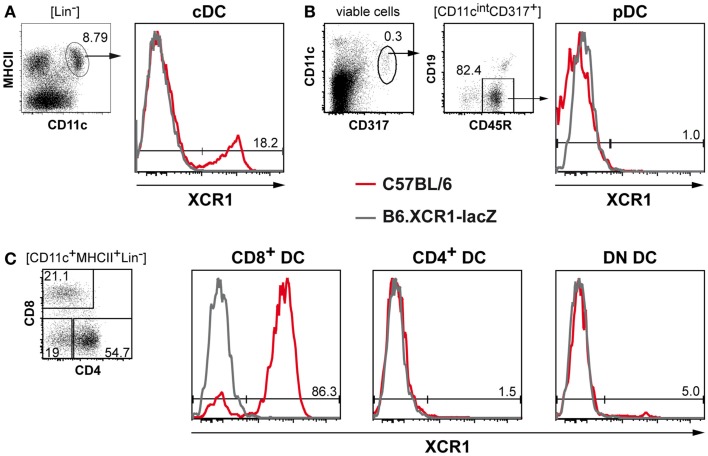
**MAb MARX10 specifically recognizes XCR1 on subsets of cDCs**. Splenocytes from C57BL/6 (red lines) or homozygous B6.XCR1-lacZ mice lacking the XCR1 receptor (gray lines) were stained with mAb MARX10 and gated on **(A)** cDCs, **(B)** pDCs, or **(C)** the cDC subsets CD8^+^, CD4^+^, and DN DCs. Shown are results representative of at least three experiments.

Immunization of homozygous B6.XCR1-lacZ reporter mice with C57BL/6 WT splenocytes enriched for CD11c^+^ DCs yielded a mAb recognizing murine XCR1. This antibody, designated MARX10, specifically stained 20% (±4% SD, *n* = 22) of splenic cDCs in C57BL/6 WT mice, but did not give a signal in homozygous B6.XCR1-lacZ animals, which lack the XCR1 receptor (Figure 1A). Similar stainings of cDCs were also obtained in BALB/c and 129/Sv animals (not shown). pDCs did not express XCR1 (Figure 1B). Negative for XCR1 were also T cells, B cells, macrophage subsets, eosinophilic, and neutrophilic granulocytes, NK cells, NKT cells, and γ/δ T cells (data not shown), as already reported at the mRNA level (Dorner et al., 2009).

### The XCR1 receptor is selectively expressed on CD8^+^ cDCs and a subset of DN cDCs

When subsets of cDCs were further analyzed based on their expression of CD4 and CD8, 83% (±6% SD, *n* = 22) of CD8^+^ DCs stained positive for XCR1, while the remaining CD8^+^ DCs were negative, as were the CD4^+^ DCs (Figure 1C). Analysis of DN cDCs consistently revealed a small but significant proportion (4 ± 1.8% SD, *n* = 19) of XCR1^+^ cells (Figure 1C). The flow cytometry data thus fully reflected the results on the expression of XCR1 obtained earlier using B6.XCR1-lacZ reporter mice (Dorner et al., 2009).

### Splenic XCR1^+^ DCs are localized in the T cell zone, the marginal zone, and the red pulp

**Figure 2:**
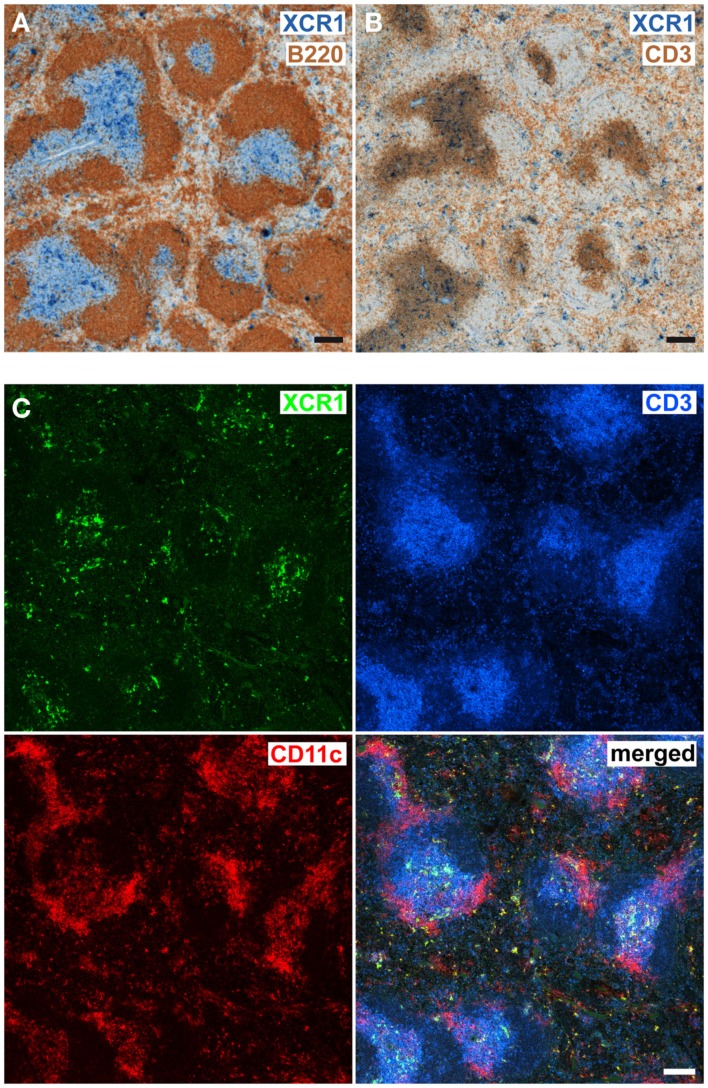
**Splenic XCR1^+^ DCs are localized in the T cell zone, marginal zone, and the red pulp**. Serial cryostat sections of C57BL/6 spleens were double-stained for **(A)** XCR1 (blue) and B220 (brown), or **(B)** XCR1 (blue) and CD3 (brown) using standard immunohistology, or **(C)** triple-stained for XCR1 (green), CD3 (blue), and CD11c (red) with fluorescent mAb and analyzed by confocal microscopy (Bars = 100 μm). Shown are representative images of two experiments.

In order to determine the anatomical localization of XCR1^+^ DCs, serial cryosections of spleens removed at steady state were stained with mAb MARX10. Signals for XCR1 were obtained in T cell zones, marginal zones, and the red pulp (Figures 2A–C), and were thus concordant with our earlier data obtained with B6.XCR1-lacZ reporter mice (Dorner et al., 2009). XCR1^+^ DCs broadly localized to the same areas as the remaining CD11c^+^ cells (Figure 2C). However, the highest density of XCR1-signals was observed in the central parts of T cell zones, while signals for CD11c^+^XCR1^−^ cells were clustered at the border (Figure 2C). Of functional importance, the location of XCR1^+^ DCs in C57BL/6 WT animals was identical to their location in *Xcl^−/−^* mice (Dorner et al., 2009 and data not shown). Moreover, the anatomical distribution of the reporter signals in heterozygous B6.XCR1-lacZ mice, which express XCR1, was identical to the signals obtained with homozygous B6.XCR1-lacZ mice, which lack the XCR1 receptor (data not shown). These results demonstrated that the positioning of splenic XCR1^+^ DCs in the T cell zone, marginal zone, and the red pulp at steady state is independent of XCR1 or its chemokine ligand XCL1.

### Expression of XCR1 on splenic cDCs is highly correlated with CD8, CD205, and Clec9A, but anti-correlated with CD4, CD172a, CD11b, CX3CR1, and DCIR2

**Figure 3:**
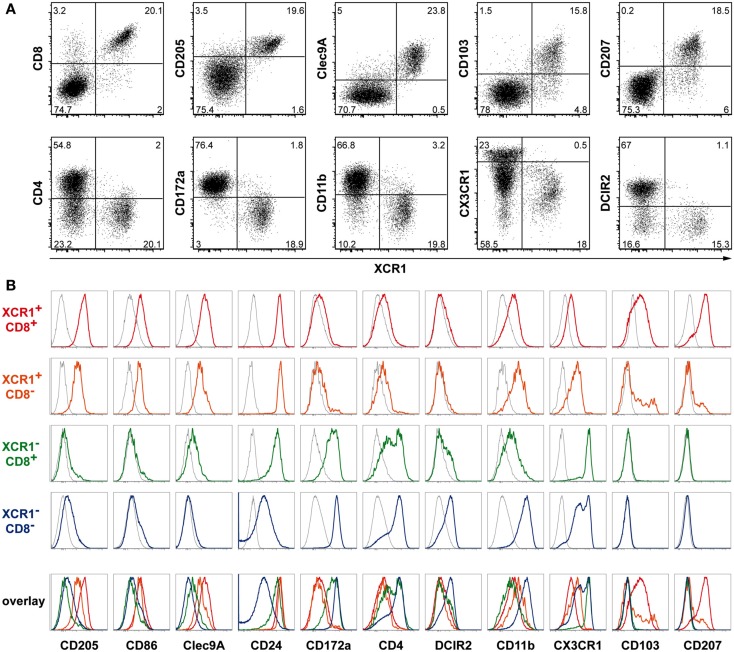
**Correlation of XCR1 expression with different DC markers**. Splenic cDCs of C57BL/6 WT, heterozygous CX3CR1^GFP^, or Lang-EGFP (CD207) mice were enriched by digestion and density gradient centrifugation and double-stained with mAb MARX10 and the indicated markers. For Clec9A/DNGR-1 staining clone 24/04-10B4 is shown, similar staining was obtained with clone 1F6. The gates were set on CD11c^+^MHCII^+^Lin^−^ cells. **(A)** Correlation and anti-correlation of XCR1 with the indicated surface molecules on splenic cDCs. Shown are results representative of three experiments. **(B)** Expression of the indicated surface markers on XCR1^+^CD8^+^ (red), XCR1^+^CD8^−^ (orange), XCR1^−^CD8^+^ (green), and XCR1^−^CD8^−^ (blue) splenic cDCs. The background staining was determined with isotype or FMO controls (gray). The data are representative of two or more experiments.

Since expression of XCR1 did not follow the subdivision of cDCs based on CD4 and CD8, we compared its expression to a greater number of other DC surface receptors. XCR1 was found to be highly correlated with CD8, CD205, and Clec9A/DNGR-1, and to a lesser degree with CD103, and CD207/langerin (Figure 3A). An anti-correlation to XCR1 expression was observed with CD4 (as described above), and interestingly also with CD172a/SIRPα, CD11b, CX3CR1/fractalkine receptor, and DCIR2 (also termed 33D1, Figure 3A).

### XCR1^+^CD8^+^ and XCR1^+^CD8^−^ DCs are related, and different from XCR1^−^CD8^+^ and XCR1^−^CD8^−^ DCs

To gain further understanding on the relationship of cells expressing XCR1, we compared splenic XCR1^+^CD8^+^ DCs (around 80% of “classical CD8^+^ DCs”) with XCR1^+^CD8^−^ DCs (around 4% of “DN DCs”). Both DC subsets expressed CD205, CD86, Clec9A, CD24, and both were lacking CD172a, CD4, and DCIR2 (Figure 3B). XCR1^+^CD8^+^ DCs exhibited lower levels of CD11b and were low/negative for CX3CR1, but their expression of CD103 and CD207 was clearly higher compared to their CD8-negative counterparts (Figure 3B). An altogether different pattern of surface receptors was observed on CD8^+^ DCs not expressing XCR1. This population was low/negative for CD205, CD103, and CD207, expressed some CD11b and Clec9A, was positive for CD172a to a substantial degree, heterogeneous for CD4, partly expressed DCIR2, and exhibited high levels of CX3CR1. Finally, XCR1^−^CD8^−^ DCs, the largest population of DCs, were low/negative for CD24, CD103, CD207, and Clec9A, expressed low levels of CD205, were largely CD4^+^, and were positive for DCIR2, CD11b, CD172a, and CX3CR1 (with a subpopulation expressing high levels of this chemokine receptor, Figure 3B). Based on this phenotypic analysis, XCR1^+^CD8^+^ DCs and XCR1^+^CD8^−^ DCs appeared related, with XCR1^+^CD8^−^ partly differing in the expression of CD103, and CD207. The phenotype of XCR1^−^ cDCs was clearly different from XCR1^+^ DCs.

### Development of splenic XCR1^+^ DCs is dependent on Flt3 ligand, Batf3, and IRF-8

**Figure 4:**
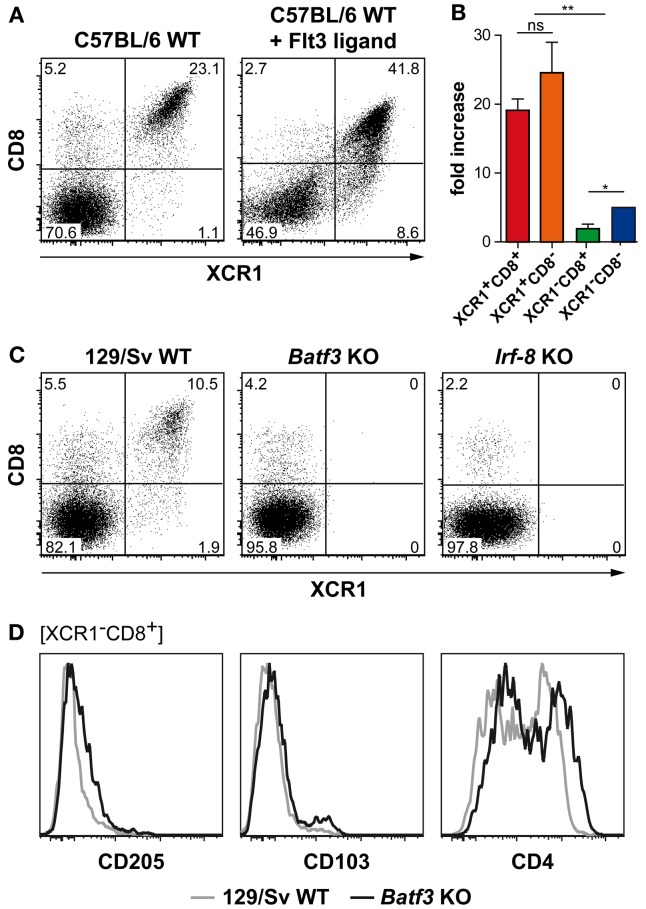
**Development of splenic XCR1^+^ DCs is dependent on the growth factor Flt3 ligand and the transcription factors Batf3 and IRF-8**. **(A,B)** C57BL/6 mice were exposed to Flt3 ligand for 9 days *in vivo*. Thereafter, spleens were removed, and the expression of CD8 and XCR1 on splenic cDCs (CD11c^+^MHCII^+^Lin^−^ cells) was compared to unexposed controls. **(A)** Shown is one representative experiment out of four. **(B)** Fold increase (based on frequencies of live) of the indicated cDC subsets in relation to unexposed controls (mean ± SEM; *n* = 4; *p < 0.05; **p < 0.01). **(C)** Splenocytes of 129/Sv WT controls, *Batf3*-deficient, or *Irf-8*-deficient mice were stained with mAbs directed to CD8 and XCR1; shown is the expression of both markers on cDCs (CD11c^+^MHCII^+^Lin^−^ cells). **(D)** Splenocytes of 129/Sv WT controls (gray lines), or *Batf3*-deficient mice (black lines) were stained for CD205, CD103, and CD4; shown are the expression profiles after gating on XCR1^−^CD8^+^ cDCs. Data are representative of two experiments.

In order to understand the ontogeny of splenic XCR1^+^ DCs, we exposed C57BL/6 WT mice to Flt3 ligand, a growth factor known to play a key role in the expansion of “classical CD8^+^ DCs” (Maraskovsky et al., 1996). Although XCR1^−^ DCs expanded to some degree (maximally sixfold), both XCR1^+^CD8^+^ and XCR1^+^CD8^−^ DCs expanded around 20-fold under the influence of Flt3 ligand *in vivo*, so they together now constituted around 50% of all cDCs, compared to 20% in untreated mice (Figures 4A,B). This observation indicated a major influence of Flt3 ligand on the ontogeny of all XCR1^+^ DCs, irrespective of their CD8 expression. Next, we examined the role of TFs known to control the ontogeny of “classical CD8^+^ DCs” (Liu and Nussenzweig, 2010; Hashimoto et al., 2011; Satpathy et al., 2011). In mice deficient for the TF Batf3 (Hildner et al., 2008) or the TF IRF-8 (also designated ICSBP, Holtschke et al., 1996), the size of the CD8^+^ DC population was clearly reduced to around 5% or less (down from 15% of 129Sv WT mice), and XCR1 could no longer be detected on these remaining CD8^+^ DCs or on any other cDCs (Figure 4C). These results indicated the requirement of Batf3 and IRF-8 in the ontogeny of XCR1^+^ DCs, irrespective of their CD8 expression. Interestingly, the residual CD8^+^ DCs in the *Batf3*-deficient animals partly expressed CD4 and were negative for CD205 and CD103, and thus corresponded to the XCR1^−^CD8^+^ DC population seen in 129Sv WT mice (Figure 4D) and C57BL/6 WT mice (Figure 3B). The observation that in *Batf3*-deficient animals all XCR1^+^ DCs were absent, while the CD8^+^ cDC population not expressing XCR1 remained unaffected, suggested that XCR1^+^ DCs specifically depend in their ontogeny on Batf3.

### Uptake of life cells is highly correlated with XCR1 expression

**Figure 5:**
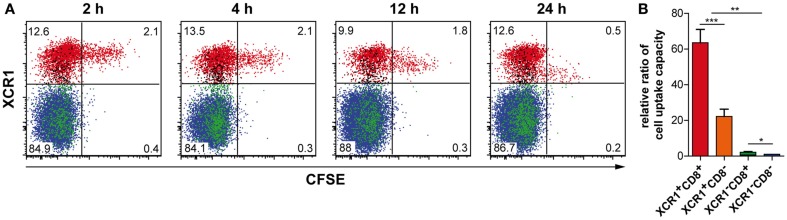
**XCR1^+^ DCs specifically take up cell-associated antigen**. C57BL/6 mice were injected with 10 × 10^6^ CFSE-labeled allogeneic 300-19-ΔOVA cells i.v., and cDCs (CD11c^+^MHCII^+^Lin^−^ cells) were analyzed for CFSE signals 2, 4, 12, and 24 h later. **(A)** The XCR1^+^CD8^+^ (red), XCR1^+^CD8^−^ (only here black!), XCR1^−^CD8^+^ (green), and XCR1^−^CD8^−^ (blue) subpopulations are color-coded. Note that XCR^+^CD8^−^ DCs express lower levels of XCR1 compared to XCR1^+^CD8^+^ DCs. Shown is one representative experiment out of three. **(B)** Relative ratio of cell uptake capacity, calculated for each animal by setting the frequency of XCR1^−^CD8^−^ DCs exhibiting a CFSE-signal to 1 and calculating the fold higher uptake for the other DC subsets accordingly (mean ± SEM of two independent experiments with three animals each; *p < 0.05; **p < 0.01; ***p < 0.001).

In order to test the function of XCR1^+^ DCs *in vivo*, we injected live, CFSE-labeled allogeneic 300-19 cells transfected with non-secretable OVA (300-19-ΔOVA) i.v., and determined their uptake by the various splenic cDC subpopulations over time. At 2 h after injection, more than 80% of the injected cells taken up by cDCs were engulfed by XCR1^+^ DCs (Figure 5A). Within the DC populations, around 13% of all XCR1^+^CD8^+^ DCs, 4% of XCR1^+^CD8^−^ DCs, 0.4% of XCR1^−^CD8^+^ DCs, and 0.2% of XCR1^−^CD8^−^ DCs have taken up 300-19 cells at 2 h, and this distribution remained similar at later time points. On a per cell basis, the capacity of these DC populations to take up the injected cells thus differed by relative ratios of 64:22:2:1 (Figure 5B). This experiment revealed a good correlation between high XCR1 expression (mainly XCR1^+^CD8^+^ DCs) and early uptake of cells. Later in the experiment, DCs which have engulfed cellular material seemed to partly downregulate XCR1. However, expression of XCR1 remained clearly detectable and that was also the case in different experiments in which DCs were activated by injection of LPS i.v. (data not shown).

### Only XCR1^+^ DCs efficiently cross-present soluble and cell-associated antigen

**Figure 6:**
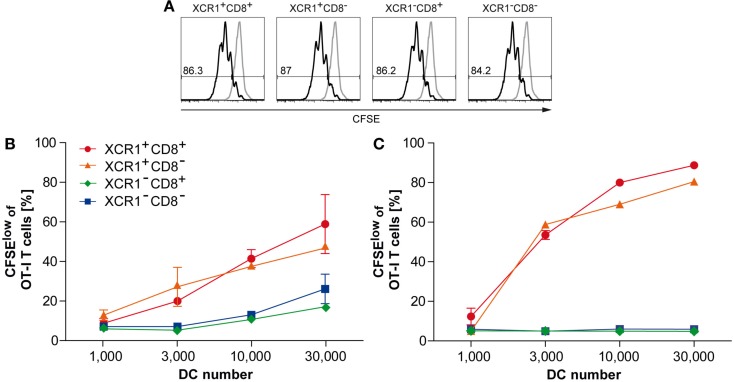
**XCR1^+^ DCs excel in cross-presentation of soluble and cell-associated antigen**. Spleens were digested, CD11c^+^ cells enriched by density gradient centrifugation and positive magnetic separation, and the XCR1^+^CD8^+^, XCR1^+^CD8^−^, XCR1^−^CD8^+^, and XCR1^−^CD8^−^ DC subsets flow-sorted to purity (≥98.5%). **(A)** The respective DC subsets were loaded with SIINFEKL peptide *in vitro* and co-cultured with CFSE-labeled OT-I T cells. Shown is the CFSE dilution profile of OT-I T cells (CD90.1^+^CD8^+^) after 60 h of co-culture with SIINFEKL-loaded DCs (black histograms) or non-loaded DC controls (gray histograms). **(B,C)** C57BL/6 mice were injected with either **(B)** 2 mg soluble OVA or **(C)** 10 × 10^6^ allogeneic cells expressing non-secretable OVA (300-19-ΔOVA cell line). Spleens were removed 14 h later and DCs were sorted to purity (≥98.5%) based on their expression of XCR1 and CD8, as above. Indicated numbers of the DC subsets were then co-cultured with 1 × 10^5^ CFSE-labeled OT-I T cells before analysis of the CSFE-dilution profile. Shown is the percentage of OT-I T cells proliferating after 60 h of co-culture with XCR1^+^CD8^+^ (red), XCR1^+^CD8^−^ (orange), XCR1^−^CD8^+^ (green), and XCR1^−^CD8^−^ DCs (blue). The error bars represent SEM. Shown are representative results of at least three experiments.

In order to test the capacity of DC subsets for antigen cross-presentation, 2 mg of soluble, endotoxin-free ovalbumin (OVA) was injected i.v. into C57BL/6 WT animals. Fourteen hours later, splenic CD11c^+^ DCs were sorted into four subsets, based on their expression of XCR1 and CD8, and co-cultured with CFSE-labeled OT-I transgenic CD8^+^ T cells at various ratios. The capacity of the DC subsets for antigen cross-presentation was determined by the degree of OT-I T cell proliferation (measured using the CFSE dilution assay) after 60 h of co-culture. Loading of the DC subsets with OVA peptide *in vitro* (without prior antigen loading *in vivo*) was used as a positive control and gave identical OT-I T cell proliferation with all DC subsets (Figure 6A). Within the DC populations loaded with OVA *in vivo*, XCR1^+^CD8^+^ DCs cross-presented soluble OVA with the same high efficiency as XCR1^+^ DCs lacking CD8 (Figure 6B). At the same time, all XCR1^+^ DCs cross-presented soluble OVA clearly better than XCR1^−^CD8^+^ and XCR1^−^CD8^−^ DCs (Figure 6B). When the experiment was repeated with cell-associated antigen (300-19-ΔOVA), XCR1^+^ DCs excelled in antigen cross-presentation, again irrespective of their CD8 expression, whereas XCR1^−^CD8^+^ and XCR1^−^CD8^−^ DCs essentially failed to cross-present OVA (Figure 6C). This dramatic difference most likely resulted from a combination of the superior ability of XCR1^+^ DCs to take up live allogeneic cells (see Figure 5), and their higher efficiency to cross-present antigen, once the cell-associated antigen has been taken up (Figure 6B). Altogether, these assays clearly demonstrated the higher capability of XCR1^+^ DCs to cross-present antigen. Interestingly, in the assays with soluble and cell-associated antigen, the smaller XCR1^+^ DC population lacking CD8, functionally behaved like the main XCR1^+^CD8^+^ DC population.

### XCR1^+^ DCs represent the Batf3-dependent lineage of DCs in peripheral lymphoid tissue and organs

**Figure 7:**
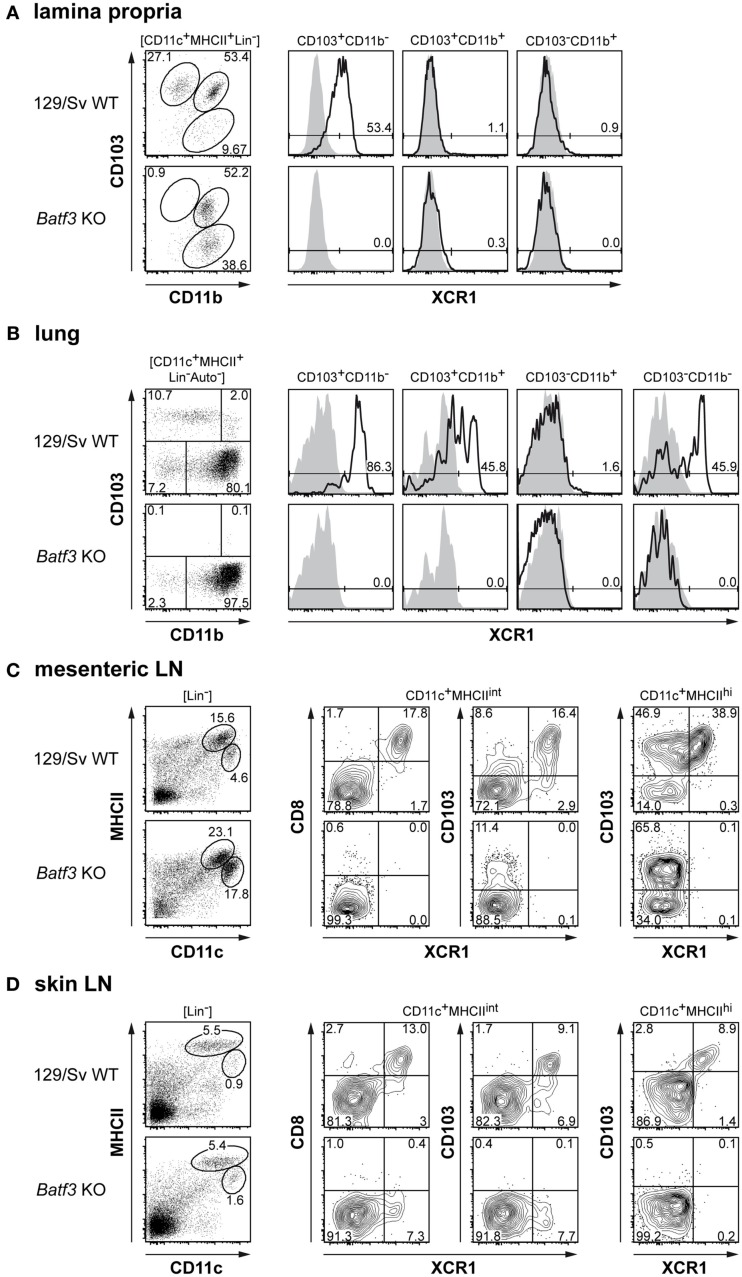
***Batf3*-deficient mice lack XCR1^+^ DCs in peripheral non-lymphoid and lymphoid tissues**. **(A)** Expression of XCR1 (black histograms) on gut lamina propria cDCs (CD11c^+^MHC II^+^Lin^−^) subdivided into CD103^+^CD11b^−^, CD103^+^CD11b^+^, and CD103^−^CD11b^+^ subsets in 129/Sv WT controls (upper row) and *Batf3*-deficient mice (lower row); the background signal (shaded histograms) was determined using homozygous B6.XCR1-lacZ mice. **(B)** Expression of XCR1 on lung cDCs (CD45^+^CD11c^+^MHC II^+^Lin^−^ autofluorescence negative) subdivided into CD103^+^CD11b^−^, CD103^+^CD11b^+^, CD103^−^CD11b^+^, and CD103^−^CD11b^−^ subsets in 129/Sv WT controls (upper row) and *Batf3*-deficient mice (lower row). **(C, D)** Presence of XCR1^+^ DCs in resident (CD11c^+^MHC^int^Lin^−^) and migratory (CD11c^+^MHCII^high^Lin^−^) cDC populations of **(C)** mesenteric or **(D)** skin-draining lymph nodes in 129/Sv WT controls (upper row) and *Batf3*-deficient mice (lower row). The results were obtained in two independent experiments. The expression of XCR1 on the various DC subpopulations in C57BL/6 mice was similar.

The similar phenotype of XCR1^+^CD8^+^ and XCR1^+^CD8^−^ splenic cDCs, their particularly strong expansion to Flt3 ligand, their specific absence in *Batf3*-deficient mice, and their superior capacity to cross-present antigen indicated that XCR1 could be a marker for a DC lineage with similar function in the entire immune system. In order to test this hypothesis, we analyzed the CD103^+^CD11b^low/−^ DCs, the equivalents of the “classical CD8^+^ DCs” in non-lymphoid peripheral organs (Shortman and Heath, 2010; Hashimoto et al., 2011) for their XCR1 expression and dependence on Batf3. In the lamina propria of the gut, these CD103^+^CD11b^−^ DCs uniformly expressed XCR1, while the CD103^+^CD11b^+^ and CD103^−^CD11b^+^ DCs were negative (Figure 7A, upper row). In *Batf3*-deficient animals this XCR1^+^ population was totally absent (Figure 7A, lower row). At the same time, the CD103^−^CD11b^+^ DC population increased in *Batf3*-deficient animals and may represent Batf3-independent precursors. In the lung, the CD103^+^CD11b^−^ DCs again uniformly expressed XCR1, but here also approximately half of the CD103^+^CD11b^+^ and half of the CD103^−^CD11b^−^ DCs were positive for XCR1, while the CD103^−^CD11b^+^ were uniformly negative (Figure 7B, upper row). Again, all XCR1^+^ lung DC populations were absent in *Batf3*-deficient animals (Figure 7B, lower row). Next, we examined LNs, where the CD11c^+^ DCs are being classified into “resident” DCs with intermediate levels of MHC class II, and “migratory” DCs with high levels of MHC class II (Ohl et al., 2004). In mesenteric LNs, the CD8^+^ DC population within the resident DCs uniformly expressed XCR1 and to a large extent overlapped with CD103^+^ DCs (Figure 7C, upper row). In *Batf3*-deficient animals, the XCR1^+^/CD8^+^ DC population was fully absent, while the XCR1^−^CD103^+^ DCs remained present (Figure 7C, lower row). Within the migratory mesenteric LN DCs, approximately half of the CD103^+^ DC population expressed XCR1, and only this population was missing in *Batf3*-deficient animals. Finally, we examined skin-draining DCs. Here, almost all of CD8^+^ DCs and approximately half of CD103^+^ DCs within the resident population were positive for XCR1 and expression of both CD8 and CD103 could no longer be detected in *Batf3*-deficient animals. However, a XCR1^+^ DC population of reduced size could still be detected, possibly representing precursors which have not yet upregulated CD8 and CD103 (Figure 7D). In the migratory population of skin-draining DCs, CD103 was highly correlated with XCR1 in WT mice, and the entire population was missing in *Batf3*-deficient animals (Figure 7D). Of note, in none of the examined tissues and LNs CD103^−^CD11b^+^ cells expressed XCR1 (not shown). When we examined IRF-8-deficient mice, XCR1^+^ DCs were absent in all described organs and LNs (including skin-draining LNs); at the same time, the lymphoid system showed additional signs of heavy disruption (not shown). Collectively, these data revealed that XCR1^+^ DCs in peripheral lymphoid organs and tissues, like their counterparts in the spleen, are in their development dependent on the TFs Batf3 and IRF-8. Even more interestingly, the results indicated that expression of XCR1 characterizes the Batf3-dependent lineage of DCs not only in the spleen, but also in peripheral lymphoid tissues and organs.

## Discussion

Since the identification of DCs in 1973 (Steinman and Cohn, 1973), many attempts have been undertaken to classify this heterogeneous cell type into subsets in order to better understand the contribution of individual DC subpopulations to antigen processing and presentation (Shortman and Heath, 2010; Steinman, 2012). Such a classification can be based on different criteria. Expression of surface proteins and secreted molecules on DCs can be considered, which together define the communication interface with other cells of the immune system. This approach is limited by the fact that many of these communication molecules are subject to up- or downregulation in the course of DC differentiation and also in the interaction with other cells. Gene expression profiling can more broadly define the functional status of DC subpopulations, but principally similar limitations apply. At the same time, gene expression profiling by itself, without meaningful preselection of cell populations, cannot identify subsets of cells within a given population. A powerful approach for the classification of DCs is the understanding of their ontogeny and differentiation (Liu and Nussenzweig, 2010; Hashimoto et al., 2011; Satpathy et al., 2011). Finally, and most importantly, classification of DCs has to be based on their particular capacity to take up, process, and present antigen.

Only very few surface molecules are in their expression restricted to cDCs, making the identification of DCs in flow cytometry or their localization in tissues inherently complex. Therefore, our previous observation that the chemokine receptor XCR1 was selectively expressed in cDCs, but not elsewhere in the body, was intriguing (Dorner et al., 2009). These observations were based on a *LacZ* XCR1-reporter mouse and qPCR analyses, but could not provide information on XCR1 surface expression. In our current work, we have generated a mAb specific for murine XCR1 allowing us to precisely determine the surface expression of the XCR1 receptor in the body. Our flow cytometry data demonstrated that XCR1 is present on around 80% of “classical CD8^+^ DCs” and on 4% of “DN” DCs in the spleen and on DC subsets in peripheral LNs and organs, but not on other cell types. Together with an extensive histological examination of tissues (data not shown), these results confirmed the DC-specific expression of XCR1.

The obtained surface expression profile of XCR1^+^ DCs could not be fully accommodated into the “classical” subdivision of splenic cDCs into CD8^+^ DCs, CD4^+^ DCs, and DN DCs (Vremec et al., 2000), raising questions about the relationship and functional relevance of DCs expressing XCR1. In our present work, we observed that DN splenic DCs expressing XCR1 (“XCR1^+^CD8^−^ DCs”) and XCR1^+^CD8^+^ DCs shared a similar phenotype, which differed from the remaining splenic DCs. Regarding their ontogeny, both XCR1^+^ DC populations were selectively expanded by the growth factor Flt3 ligand, which earlier has been shown to specifically expand “classical CD8^+^ DCs” (Maraskovsky et al., 1996). Both XCR1^+^CD8^+^ and XCR1^+^CD8^−^ DCs were missing in mice deficient for the TF IRF-8 and also in mice deficient for the TF Batf3, a model system in which 70–90% of CD8^+^ DCs are absent and in which antigen cross-presentation is severely impaired (Hildner et al., 2008; Bar-On et al., 2010). In terms of function, only the two splenic XCR1^+^ DC subsets were capable of phagocytosing allogeneic cells injected into the circulation, as has been originally demonstrated for “classical CD8^+^ DCs” (Iyoda et al., 2002). Finally, and most importantly, only XCR1^+^ DCs (irrespective of their CD8 expression) were efficient in the cross-presentation of soluble or cell-associated antigen, while CD8^+^ DCs not expressing XCR1 fully failed.

In recent years, evidence accumulated that there is no strict correlation between CD8 expression and cross-presentation, since splenic CD8^+^ cDCs were recognized as heterogeneous and also some CD8^−^ DCs were shown to have cross-presenting capacity. Lin et al. (2008) noticed that injection of cytochrome *c* depleted a proportion of CD8^+^ DCs, most strongly affecting the ability to cross-present cell-associated antigen *in vivo*. Qiu et al. (2009) determined that uptake of allogeneic apoptotic cells, as well as cross-presentation of soluble and cell-associated antigen, is strongly associated with CD8^+^ DCs co-expressing CD103 and CD207, but not with CD8^+^ DCs lacking these two molecules. Farrand et al. (2009) also found that only CD207^+^CD8^+^ DCs, but not CD207^−^CD8^+^ DCs could cross-present soluble antigen injected *in vivo*. Bar-On et al. (2010) segregated CD8^+^ DCs based on their expression of CX3CR1, the fractalkine receptor. CX3CR1^+^CD8^+^ DCs, in contrast to CX3CR1^−^CD8^+^ DCs, were not expanded by Flt3 ligand, were not affected in *Batf3*-deficient mice, were spared from the effect of injected cytochrome *c*, and did not secrete IL-12p70 upon stimulation with CpG. The authors did not perform any antigen (cross) presentation experiments, but concluded on the basis of their data that CX3CR1^+^CD8^+^ DCs lack all hallmark features of “classical CD8^+^ DCs.”

When comparing our phenotypic and functional data on splenic DCs with the results of the groups outlined above, it is apparent that the XCR1^−^CD8^+^ DCs, still present in *Batf3*-deficient animals and incapable of antigen cross-presentation, correspond to the splenic CD8^+^ DC population more extensively described by Bar-On et al. (2010). Further comparison clearly shows that the XCR1^+^CD8^+^ DC population corresponds to CD8^+^ DCs expressing CD205, Clec9A, CD103, CD207, but lacking CX3CR1. Thus, within the CD8^+^ DCs, only the XCR1^+^CD8^+^ DCs truly represent the “classical CD8^+^ DCs” described to phagocytose apoptotic cells (Iyoda et al., 2002), to cross-present antigen (den Haan et al., 2000; Pooley et al., 2001), and to secrete IL-12p70 (Hochrein et al., 2001).

Regarding the XCR1^+^CD8^−^ DCs (XCR1^+^ “DN” DCs), Vremec et al. (2007) described DN DCs characterized as highly positive for CD24 but lacking CD172a, and having some cross-presentation capability *in vitro* and *in vivo*. Bedoui et al. (2009) characterized a CD8^−^ cDC population expressing CD24 but lacking CD11b and CD172a, which was substantially expanded by Flt3 ligand *in vivo* and showed cross-presentation activity after loading with antigen *in vitro*. This population, when adoptively transferred into syngeneic hosts, acquired the CD8 surface marker over the course of several days, allowing the authors to conclude that these DCs are precursors of CD8^+^ DCs. Considering these phenotypic and functional data, it is quite apparent that the DC population described by both groups are the XCR1^+^CD8^−^ DCs.

In the spleen thus only CD8^+^ DCs expressing XCR1 on the surface correspond to the “classical CD8^+^ DCs” known to cross-present antigen. XCR1^+^CD8^−^ DCs, a subpopulation of “classical DN DCs,” are apparently precursors of XCR1^+^CD8^+^ DCs, which have not yet acquired the final surface density of XCR1 and have not yet upregulated CD103 and CD207. These XCR1^+^CD8^−^ DCs are not yet fully efficient in the uptake of cell-associated antigen, but are already endowed with the capacity to cross-present soluble antigen *in vivo*. The cross-presentation capacity in the spleen is thus provided collectively by XCR1^+^CD8^+^ and XCR1^+^CD8^−^ DCs.

In peripheral lymphoid organs and tissues, identification of “CD8^+^ DC equivalents” remained difficult. CD8, although present on resident DCs in peripheral LNs, is not clearly expressed on migratory DCs in LNs or on DCs in tissues, and thus could not be universally used as a marker. Instead, a variety of other surface molecules, among them CD103, CD11b, CD172a, F4/80, and CX3CR1 were used in attempts to demarcate the “CD8^+^ DC equivalents” in tissues other than spleen. These approaches revealed that CD103^+^CD11b^−^ DCs share a specific gene expression pattern with splenic “classical CD8^+^ DCs” (Edwards et al., 2003; Edelson et al., 2010; Crozat et al., 2011), and are dependent on the TFs Batf3, IRF-8, and Id2 (Schiavoni et al., 2002; Aliberti et al., 2003; Hildner et al., 2008; Ginhoux et al., 2009; Edelson et al., 2010). As a result, “CD103^+^CD11b^−^ DCs,” or alternatively “Batf3-IRF-8-Id2-dependent DCs” are currently being regarded as peripheral equivalents of “classical CD8^+^ DCs” (Shortman and Heath, 2010; Hashimoto et al., 2011). Based on a phenotypical analysis of XCR1-reporter mice and gene expression studies, Crozat et al. (2011) recently proposed that CD8α^+^ and CD103^+^ DCs belong to a common DC subset characterized by XCR1 expression.

In our work on peripheral DCs, expression of XCR1 coincided with the presence of CD8 on LN resident DCs, and was highly correlated with CD103^+^CD11b^−^ DCs in all tissues. However, in some tissues also about half of CD103^+^CD11b^+^ and CD103^−^CD11b^−^ DCs expressed XCR1 on the surface. Thus, XCR1^+^ DCs in the periphery could not be easily accommodated into the current definition of “CD8^+^ DC equivalents” based on the expression of CD103 and CD11b only. Interestingly, however, peripheral XCR1^+^ DCs were uniformly missing in *Batf3*-deficient mice (with the partial exception of skin-draining DCs), while substantial populations of CD103^+^ DCs remained present. Altogether, our results revealed an almost perfect correlation between Batf3-dependency and XCR1 expression for DCs in various lymphoid and non-lymphoid organs in the periphery. In IRF-8-deficient mice, XCR1^+^ DCs were missing, but also additional immune populations were absent (data not shown).

The consistent and apparently selective absence of XCR1^+^ DCs, both in the spleen and the periphery of *Batf3*-deficient animals, strongly indicates that expression of XCR1 stringently characterizes the Baf3-dependent lineage of DCs in the immune system. None of the current surface molecules used to define “CD8-type” cross-presenting DCs shows this specificity. Both CD8 and the integrin CD103 are expressed on Batf3-independent DCs which are incapable of antigen cross-presentation, and in addition on other cell types in the immune system (Heath and Carbone, 2009; Hashimoto et al., 2011). CD205, also functionally linked to cross-presentation in splenic cDCs (Jiang et al., 1995; Dudziak et al., 2007), is also expressed on B cells, epithelial cells, and stromal cells (Inaba et al., 1995; Witmer-Pack et al., 1995). Clec9A is correlated with XCR1 in the spleen, but is also found on splenic XCR1^−^CD8^+^ DCs (this work and Caminschi et al., 2012), on pDCs, and a subset of B cells (Caminschi et al., 2008; Sancho et al., 2008). Thus, expression of XCR1 uniquely defines a lineage of DCs with apparently similar function throughout the immune system. This correlation of XCR1 expression with DC function will facilitate future analysis of DC biology in the mouse and potentially also in the human.

Based on our work in the mouse and on the subsequent recognition that peripheral blood XCR1^+^ DCs are the human homologs of murine “classical CD8^+^ DCs,” we (Dorner et al., 2009; Bachem et al., 2010) and others (Crozat et al., 2010) have proposed that targeting of antigen to XCR1^+^ DCs *in vivo* may be a valid option to induce CD8^+^ T cell cytotoxic immunity against pathogens or tumors in man. With the recognition that XCR1 is selectively expressed on DCs specialized on antigen cross-presentation in the entire immune system, this notion becomes even more attractive.

## Conflict of Interest Statement

The authors declare that the research was conducted in the absence of any commercial or financial relationships that could be construed as a potential conflict of interest.

## Author Note

During the submission process of this manuscript we became aware of the work of L.F. Poulin et al. (Blood online prepublication, doi: 10.1182/blood-2012-01-406967), in which the authors describe DNGR-1/Clec9A as a specific marker for mouse and human Batf3-dependent DCs. In our own work, expression of Clec9A on splenic cDCs was highly congruent with XCR1. A congruence of Clec9A and XCR1 expression may also be true in peripheral organs, but due to the low signal intensity of Clec9a staining in peripheral tissues we could not assess any correlation there. However, there is a clear difference between these two surface receptors. Clec9A is also expressed on murine pDCs and on a small subset of B cells (Caminschi et al., 2008; Sancho et al., 2008), populations which are independent of Batf3 in their ontogeny (Hildner et al., 2008), while XCR1 is exclusively expressed on cDCs. We thus conclude that only expression of XCR1 closely reflects the Batf3-dependent lineage of murine cDCs.

## Abbreviations

cDC, conventional DC; DIG, digoxigenin; DN, double negative; FMO, fluorescence minus one; pDC, plasmacytoid DC; TF, transcription factor.
